# Graphene-Induced Osteogenic Differentiation Is Mediated by the Integrin/FAK Axis

**DOI:** 10.3390/ijms20030574

**Published:** 2019-01-29

**Authors:** Han Xie, Tong Cao, Alfredo Franco-Obregón, Vinicius Rosa

**Affiliations:** 1 Faculty of Dentistry, National University of Singapore, 9 Lower Kent Ridge Road, Singapore 119085, Singapore; xiehan512@foxmail.com (H.X.); dencaot@nus.edu.sg (T.C.); 2 Department of Surgery, Yong Loo Lin School of Medicine, National University of Singapore, NUHS Tower Block, Level 8, IE Kent Ridge Road, Singapore 119228, Singapore; suraf@nus.edu.sg; 3 BioIonic Currents Electromagnetic Pulsing Systems Laboratory, BICEPS, National University of Singapore, MD6, 14 medical Drive, #14-01, Singapore 117599, Singapore; 4 Department of Materials Science and Engineering, National University of Singapore, Blk EA, #03-09 9 Engineering Drive 1, Singapore 117575, Singapore; 5 Centre for Advanced 2D Materials and Graphene Research Centre, National University of Singapore, 6 Science Drive 2, Singapore 117546, Singapore

**Keywords:** tissue engineering, differentiation, focal adhesion kinase, bone, nanomaterials, osteoblast, mechanical

## Abstract

Graphene is capable of promoting osteogenesis without chemical induction. Nevertheless, the underlying mechanism(s) remain largely unknown. The objectives here were: (i) to assess whether graphene scaffolds are capable of supporting osteogenesis in vivo and; (ii) to ascertain the participation of the integrin/FAK mechanotransduction axis during the osteogenic differentiation induced by graphene. MSC-impregnated graphene scaffolds (*n* = 6) were implanted into immunocompromised mice (28 days). Alternatively, MSCs were seeded onto PDMS substrates (modulus of elasticity = 130, 830 and 1300 kPa) coated with a single monomolecular layer of graphene and cultured in basal medium (10 days). The ensuing expressions of FAK-p397, integrin, ROCK1, F-actin, Smad p1/5, RUNX2, OCN and OPN were evaluated by Western blot (*n* = 3). As controls, MSCs were plated onto uncoated PDMS in the presence of mechanotransduction inhibitors (echistatin, Y27632 and DMH1). MSC-impregnated graphene scaffolds exhibited positive immunoexpression of bone-related markers (RUNX2 and OPN) without the assistance of osteogenic inducers. In vitro, regardless of the stiffness of the underlying PDMS substrate, MSCs seeded onto graphene-coated PDMS substrates demonstrated higher expressions of all tested osteogenic and integrin/FAK proteins tested compared to MSCs seeded onto PDMS alone. Hence, graphene promotes osteogenesis via the activation of the mechanosensitive integrin/FAK axis.

## 1. Introduction

Graphene consists of a single sheet of carbon atoms arranged into a tightly packed two-dimensional (2D) honeycomb lattice. Graphene sheets have proven quite amenable to molecular chemical vapor deposition functionalization as well as present high specific surface area and ultrahigh mechanical strength. Graphene can also be produced by (CVD) that is a scalable method to produce high quality graphene substrates in two and three-dimensions [1].

The demonstrated ability of CVD-grown graphene to induce osteogenic differentiation in vitro has rendered this method as a promising platform for cell-based bone tissue engineering and regeneration [2,3,4,5,6,7,8,9]. Notably, stem cells cultured on graphene films with basal growth medium demonstrated elevated expression of osteogenic proteins including Runt-related transcription factor 2 (RUNX2), osteocalcin (OCN), alkaline phosphatase (ALP), and osteopontin (OPN) [2,4,8]. Similarly, three-dimensional graphene substrates (scaffolds and hydrogels) increased the expression of bone-related genes and proteins such as RUNX2, collagen I (COL-1), and OCN [4,10,11]. Graphene film has also been shown to augment the mineralization potential of different somatic and stem cell classes [2,3,7]. Remarkably, MG-63 cells cultured on graphene coated titanium exhibited higher amounts of calcium nodules after 7 days compared to the uncoated controls (12 vs. 8 Ca/ng of DNA) [7]. The osteogenic differentiation achieved with CVD-grown graphene in the absence of osteogenic inductors is highly advantageous for bone tissue engineering as these chemicals and biomolecules present the following shortcomings: (a) dexamethasone is relatively non-specific promoting both osteogenesis as well as adipogenesis in the long-term; (b) bone morphogenetic proteins (BMPs) are expensive and plagued by concerns over their safety and; (c) the systematic administration of glucocorticoids can paradoxically result in bone loss [12,13,14].

Although graphene has been shown to induce osteogenic differentiation in vitro the intracellular events involved in this phenomenon remain largely unresolved. The physical characteristic of graphene (elastic properties and superficial topographical features such as wrinkles and ripples) may facilitate cell anchorage and thereby, promote stem cell differentiation [2,8]. Previous studies have shown that mesenchymal stem cells (MSCs) undergoing osteogenic differentiation on graphene in the absence of osteogenic inductors, exhibiting an upregulation in the basal expression of collagen I, RUNX2, OCN and OPN as well as other osteogenic genes [2,4,6,10,15]. Graphene activates physiologically-relevant mechanotransduction pathways, stimulating the expression of transcripts for bone morphogenetic protein 2 (BMP-2) and myosin heavy chain [4,6] and supported by findings that myosin heavy-chain mRNA and protein are upregulated by mechanical loading [16]. NIH-3T3 fibroblasts cultured on graphene coated substrates (glass, silicone or polydimethylsiloxane) exhibited enhanced adhesion and spreading and higher expression of F-actin and vinculin. Moreover, fibroblasts grown on graphene exhibited more elaborated stress fiber cytoarchitecture than those fibroblasts grown on uncoated substrates [17]. Cells grown on graphene films or scaffolds exhibited greater activation of focal adhesion kinase (FAK) [17,18], a key component of focal adhesion-mediated mechanotransduction regulating proliferation, differentiation and apoptosis, and other key processes in cellular physiology [19]. Finally, FAK is an essential component of the signal transduction machinery controlling osteogenesis, whereby its suppression disrupts bone formation [20]. 

Stem cell lineage commitment is modulated by the elastic characteristics of their microenvironments [21]. For instance, MSCs cultured in a rigid hydrogel (G’ = 3600 Pa) exhibited higher mineralization potential than those cultured in hydrogels with elastic modulus of G’ ≤ 1570 Pa [22]. Similarly, stem cells from human exfoliated deciduous teeth (SHED) exhibit greater potential for osteogenic differentiation with increasing substrate rigidity [23]. Substrate matrix stiffness influences osteogenic differentiation of MSC via mechanosensing pathways conveyed through α-integrins, Rho-associated protein kinase (ROCK) and focal adhesion kinase (FAK) [24,25]. Indeed, an increase in the substrate stiffness enhances osteoblastic differentiation through the α2-integrin-ROCK-FAK-ERK1/2 axis [26]. As graphene has a very high elastic modulus (>1.0 TPa [27]), high in-plane stiffness [3] and remarkable flexibility for out-of-plane deformation [8], it is reasonable to assume that the osteogenic differentiation promoted by this material is similarly triggered and mediated by the activation of the mechanotransduction integrin-FAK axis. Unveiling the intracellular cascade(s) evoked by graphene and governing osteogenic differentiation is thus critical for the effective clinical translation of this carbonaceous and versatile material.

The objectives of this study were: (i) to assess the ability of a graphene scaffold to promote osteogenic differentiation of human MSCs in vivo and; (ii) to test the hypothesis that the osteogenic differentiation of MSCs on graphene is mediated via the activation of the integrin-FAK axis.

## 2. Results

### 2.1. Graphene Scaffold Induced Osteogenic Differentiation In Vivo

**Figure 1:**
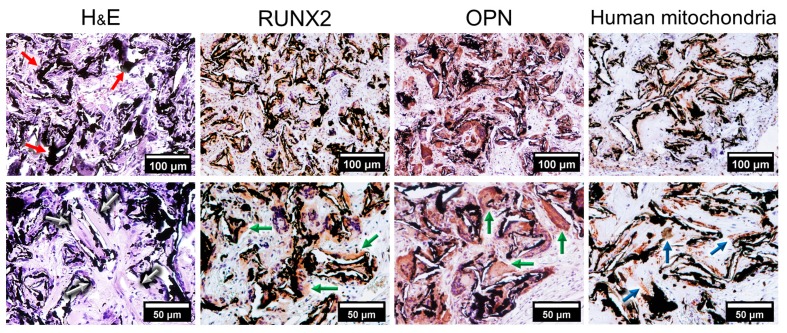
Graphene scaffold induce osteogenic differentiation in vivo. MSCs were seeded onto graphene scaffolds and transplanted into subcutaneous pockets of SCID mice. After four weeks, there was the formation of bony-like structures on the surface of the graphene scaffold (H&E, white arrows) that presented positive expression for the markers of osteogenic differentiation Runt-related transcription factor 2 (RUNX2) and osteopontin (OPN) (green arrows). The positive staining for specific for human mitochondria confirmed that the tissues formed were populated by human cells (blue arrows).

To evaluate whether graphene was capable of inducing osteogenesis in vivo we seeded MSCs onto a graphene scaffold (red arrows in H&E) before transplantation into the subcutaneous space of SCID mice for four weeks (Figure 1). The histological sections obtained from the scaffolds revealed the formation of bony-like structures resembling primary bone (white arrows in H&E). Immunohistochemical characterization showed that the tissues presented positive expression of the bone-related markers RUNX2 and OPN (green arrows) and for specific human mitochondria antibody (blue arrows).

### 2.2. Activation of Integrin-FAK Axis during Graphene-Mediated Osteogenic Differentiation

To determine whether graphene promotes osteogenic differentiation via the actions of mechanosensitive pathways, we analyzed the expression of key proteins involved in the integrin-FAK axis in response to substrates of diverse mechanical properties. MSCs were seeded onto PDMS substrates with tuned elastic moduli (PDMS) or graphene-coated PDMS (Gp) in the presence or absence of inhibitors. The expression of the proteins was evaluated after 10 days in vitro.

**Figure 2:**
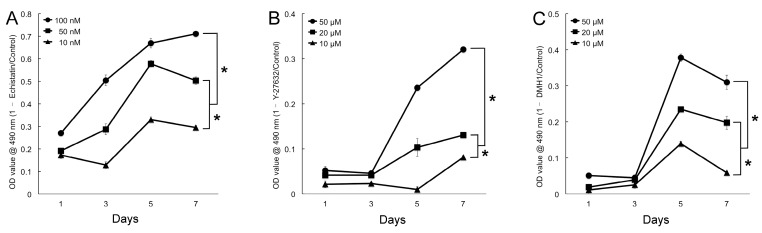
Effects of mechanotransduction inhibitors on cell proliferation. All inhibitors concentrations decreased cell proliferation at all time points compared to controls. After seven days, the proliferation decreased by approximately 30%, when cells were treated with 10 nM of echistatin (**A**) and 50 µM of Y27632 and DMH1 (**B**,**C**) comparing to the untreated control. (* denotes statistical difference between the groups, *p* < 0.05. For the sake of clarity, only the statistical significances at day seven are depicted).

The effective concentrations of inhibitors were ascertained by their ability to suppress proliferation. MSCs were administered different concentrations of echistatin (a Disintegrin), Y-27632 (p160ROCK inhibitor) or DMH1 (Activin receptor-like kinase 2 inhibitor) and their growth monitored for seven days. A decrease in cell proliferation of approximately 30% compared to the control was set as the threshold for effective inhibition. Echistatin significantly inhibited proliferation as early as 1 day (*p* < 0.05) at all concentrations used. However, at 10 nM proliferation was reduced by approximately 30% after seven days (Figure 2A). Effective proliferation inhibition was obtained at a concentration of 50 µM for both Y27632 and DMH1 (Figure 2B,C).

**Figure 3:**
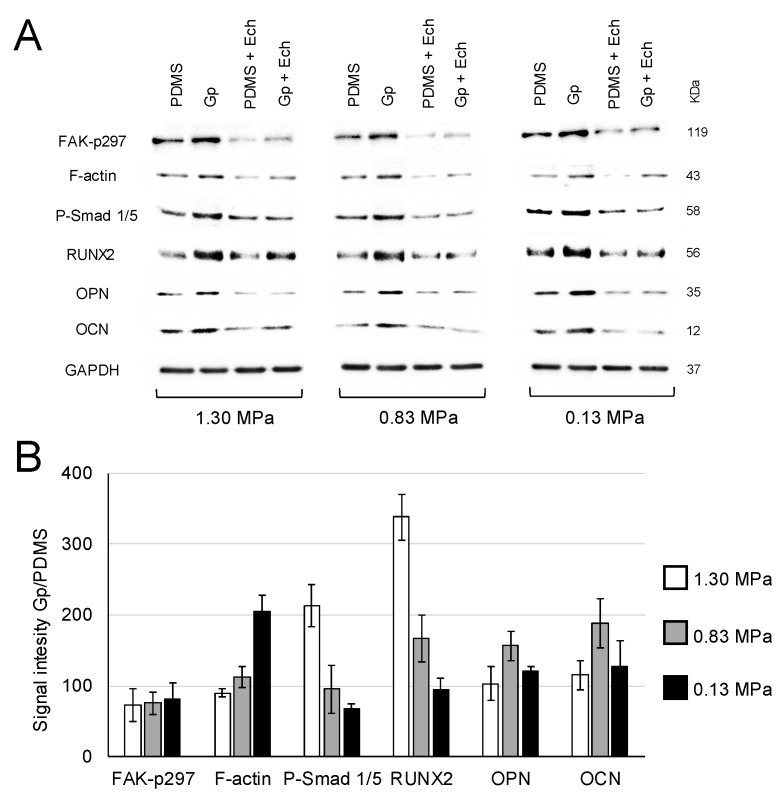
(**A**) Absolute and (**B**) relative expression levels of indicated proteins derived from MSCs grown on PDMS of different stiffnesses (determined by ratio of Sylgard 184 and 527) and graphene-coated PDMS (Gp). Regardless of the stiffness of the underlying substrates, MSC on Gp presented higher expression of physical stimuli-related proteins (FAK-p397, Smad p1/5 and F-actin) and bone-related markers (RUNX2, osteopontin (OPN) and osteocalcin (OCN)) compared to cells cultured on PDMS alone. OPN and OCN expression increased on Gp relative to PDMS (Gp/PDMS) for all stiffnesses tested. (**B**) relative quantification of all groups in the absence of inhibitors. Signal intensity is in arbitrary units. The presence of 10 nM echistatin attenuated the expression of all proteins examined. GAPDH represents housekeeping gene.

Next, we evaluated whether the integrin-FAK axis was activated during graphene-induced osteogenic differentiation. MSCs were cultured on PDMS substrates of varying stiffness that had been coated with a single monomolecular layer of graphene (Gp), or not. After 10 days, MSCs grown on Gp presented higher expression levels of FAK-p397, as well as all downstream proteins recruited in this axis compared to those seeded on PDMS alone. Highest expressions were observed on graphene-coated substrates (Gp) regardless of the stiffness of the underlying PDMS substrate. The expression of all mechanotransductory-related proteins was decreased by the presence of Echistatin (10 nM), strongly implicating the integrin-FAK axis in the osteogenic differentiation triggered by graphene (Figure 3A,B). The quantification of relative expressions showed that cells grown on Gp exhibited higher protein expression than cells cultured on PDMS alone of similar modulus of elasticity (Figure 3B).

**Figure 4:**
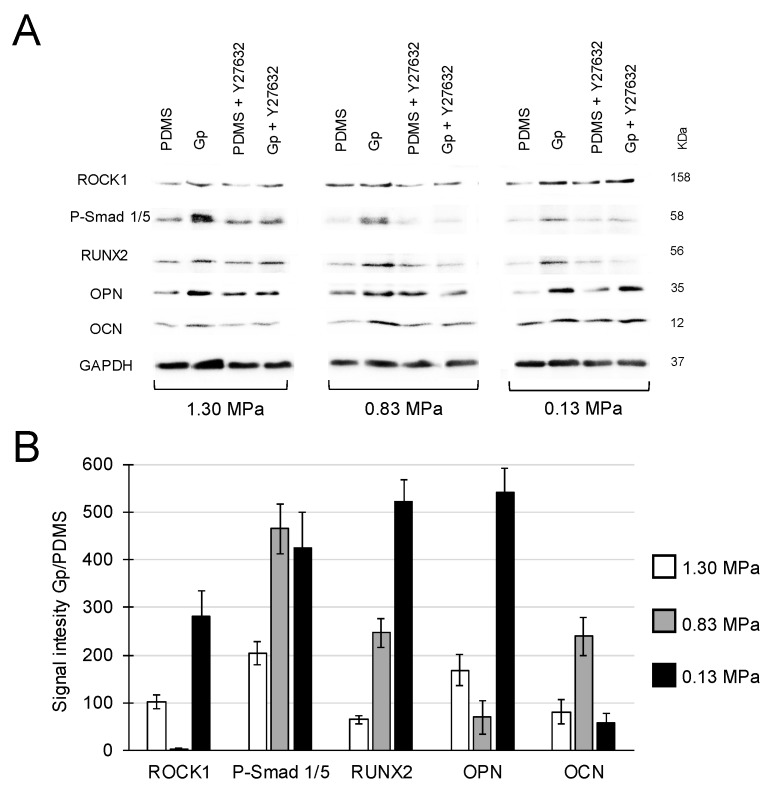
(**A**) Regardless of the stiffness of the underlying substrate (PDMS), Gp upregulated the expression levels of ROCK1, Smad p1/5 and F-actin and bone-related proteins. With the exception of ROCK1/0.83 MPa, Gp increased the expression of all proteins by >50%. (**B**) Relative quantification of all groups in the absence of inhibitors. Signal intensity is in arbitrary units.

Y27632 (50 μM) was used to confirm a downstream role of ROCK1 in the osteogenic differentiation induced by graphene. As previously, regardless of the stiffness of the underlying polymer, MSCs on graphene-coated PDMS exhibited higher expression levels of ROCK1 in conjunction with its downstream affiliated transforming growth factor β modulating protein, Smad 1/5, and bone-related proteins (RUNX2, OPN and OCN), whose expressions were attenuated by the administration of Y27632 (Figure 4A,B).

**Figure 5:**
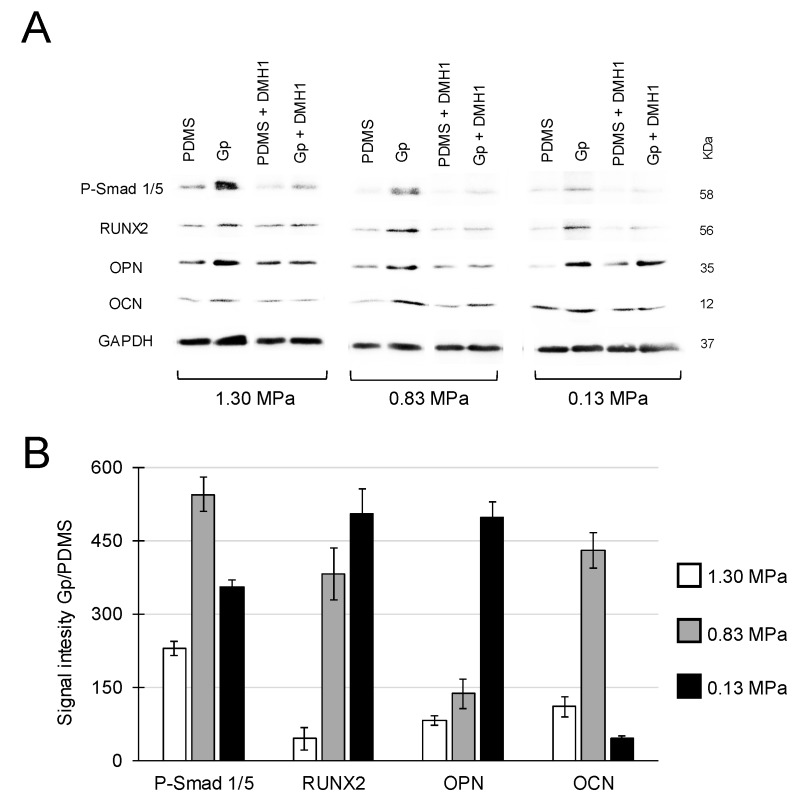
(**A**) MSCs grown on Gp exhibited greater increases of Smad p1/5 and of the classical markers for osteogenic differentiation, RUNX2, OPN and OCN. The expressions of p-SMAD increased by >180% on Gp compared to PDMS alone. The treatment 50 µM of DMHI decreased the expression of all protein studies. (**B**) Relative quantification of all groups in the absence of inhibitors. Signal intensity is in arbitrary units.

Finally, we checked the expression levels of the selected proteins before and after inhibiting Smad p1/5 in response to treatment with DMH1 (50 µM). The expressions of Smad p1/5 and of the downstream bone-related proteins (RUNX2, OPN and OCN) were higher on Gp compared to all PDMS conditions tested. The presence of DMH1 suppressed the expression of all proteins confirming that the osteogenic differentiation on graphene is regulated by the activation of Smad p1/5 (Figure 5A). The quantification of protein expression showed that cells on Gp exhibited increased compared to PDMS for all modulus of elasticities studied (Figure 5B).

## 3. Discussion

CVD-grown graphene has emerged as a promising platform with which to enhance osteogenic outcome [5,6,8,9,23]. The osteogenic capabilities of CVD-grown graphene have been attributed to its molecular structure, whereby the potential for π–π stacking between the aromatic rings of given osteogenic inducers (e.g., dexamethasone, β-glycerophosphate) and the graphene has been hypothesized. This would act to effectively absorb and immobilize said biomolecules to its surface for more effective cell delivery and enhanced differentiation [3,24]. Seemingly at odds with the previous studies, several other publications have attested to the osteogenic potential of graphene films, scaffolds and hydrogels even in the absence of chemical inductors [2,4,6,8,12]. A resolution to these seemingly disparate discoveries may come with the recent finding that unstimulated paracrine cells plated onto graphene enhance their release of signaling molecules and proteins that are capable of promoting osteogenic differentiation of MCSc cultured onto inert glass substrate [2]. Hence, it is feasible that graphene both promotes the paracrine release of pro-osteogenic biomolecules in the immediate microenvironment as well as enhances their delivery to support osteogenic differentiation [2,4,8,11]. However, the potential of CVD-grown graphene promote osteogenesis in vivo and the cellular mechanisms evoked to allow differentiation remain largely unknown. In this report, the graphene scaffold induced the differentiation of human MSC in vivo (Figure 1). After four weeks from implantation, the histological analysis with H&E revealed the presence of bony-like tissues within the scaffolds resembling primary bone. Notably, the imunnohistochemical analysis of the tissues formed showed positive expression of the transcription factor RUNX2 and OPN. The first is essential for osteoblastic differentiation and skeletal morphogenesis, while OPN is an important bone matrix protein synthesized by osteoblastic cells [25,26]. Positive immunoreactivity to an antibody specific for human mitochondria indicated that the tissues formed was of human origin and confirmed that the cells remained viable for 28 days as well as that they underwent osteogenic differentiation in vivo.

Evidence exists for integrin-mediated mechanical signaling regulating stem cell paracrine factor production and release [10]. We investigated the role of the integrin-FAK-axis in the graphene-induced osteogenic differentiation. The FAK activity is related to alterations in the actin and microtubule filaments. As cells experience changes in forces through integrin contacts that link the extracellular matrix with the cytoskeleton, the FAK is activated modulating corrective cell responses to environmental stimuli [18,27]. Both integrins and FAK proteins are sensitive to physical stimuli and elastic properties of the substrates [28]. Accordingly, the high modulus of elasticity (up to 2.4 TPa) and high flexibility to out-of-plane deformation of graphene [8,22] could modulate the expression of these proteins and trigger a mechano-stimulated osteogenic differentiation. To test this hypothesis, MSC were cultured on PDMS substrates with modulus of elasticity ranging from 130 to 1.3 MPa with or without a coating of graphene. After 10 days, the expressions of the proteins involved in the integrin-FAK axis were evaluated by Western blot.

The expression of FAK-p397 was higher in all graphene-coated PDMS substrates (Gp) compared to PDMS alone (Figure 3). Similar enhancement in FAK expression was observed in fibroblasts cultured on stiff fibronectin gel (66 kPa) compared with a softer one (1050 Pa) [29]. Besides regulating cell adhesion, FAK-p397 is necessary for osteogenic differentiation, as its inhibition decreases RUNX2 expression by ~50% and mineralized matrix deposition by 30% [30].

Additionally, we observed that MSCs on Gp presented higher F-actin expression compared MSCs on uncoated PDMS (Figure 3). F-actin controls the cytoskeleton tension, promotes the phosphorylation of Smad and upregulates the downstream of osteogenic regulatory proteins [31]. The higher expression of RUNX2, OCN and OPN observed in this study may be related to greater substrate-mediated contractile forces reflected by increased F-actin stress fibers that enhances osteoblastic differentiation [18]. In addition, regardless of the stiffness of the underlying PDMS and presence of graphene, cells treated with the integrin inhibitor (echistatin) presented low expression of all proteins analyzed. This inhibitor binds to integrin α_v_β_3_ receptor in a nondissociable manner [32] decreasing the expression of FAK, actin cytoskeleton and RhoA, hence, compromising the integrin-FAK signaling [33]. A similar trend ca be observed in Figure 3, confirming that the integrin-FAK complex is triggered by graphene, inducing osteogenic differentiation.

Contractile and mechanical forces associated by stiffer substrates activate RhoA/Rho-associated protein kinase (ROCK) that is essential for the formation of stress fibers and for the activation of Smad signaling imperative for osteogenesis [34,35]. MSCs on Gp exhibited higher expression of ROCK1, Smad p1/5 and both OCN and OPN compared to MSCs seeded onto PDMS alone (Figure 4). The latter are well-recognized signature proteins for the presence and function of mature osteoblasts [2,4]. Conversely, treatment with Y27632 decreased the expressions of ROCK1, Smad p1/5 and bone-related markers. Moreover, periodontal ligament cells treated with Y27632 also showed significant reductions in the expression of osteogenic genes (e.g., fibronectin 1, collagen type I and III, and biglycan) [36]. Together, these findings confirm that the graphene-induced osteogenic differentiation of MSC is mediated by the expression of ROCK1.

Finally, we checked the role of Smad (Figure 5), which is a sensitive regulatory protein that mediates the osteogenic differentiation in MSCs [37]. A single layer graphene increased the expression of Smad p1/5 independently of the stiffness of the underlying PDMS substrate. It is known that the phosphorylated Smad protein complex (e.g., Smad p1/5/8) can translocate into the cell nucleus and directly induce an upstream activator of RUNX2 [37,38], while the suppression of phosphorylation and nuclear translocation of Smad 1/5/8 inhibits the expression of osteogenic genes in MSCs [39]. Smad functions with RUNX2 through direct binding in a transcriptional activator complex during the osteogenic induction [40]. Accordingly, MSCs grown on Gp revealed the increased expression of Smad p1/5 and RUNX2 culminating in higher expressions of OCN and OPN proteins. The expressions decreased by the presence of DMH1 inhibitor confirming that the graphene-induced osteogenic differentiation is regulated by the upstream of Smad proteins.

**Figure 6:**
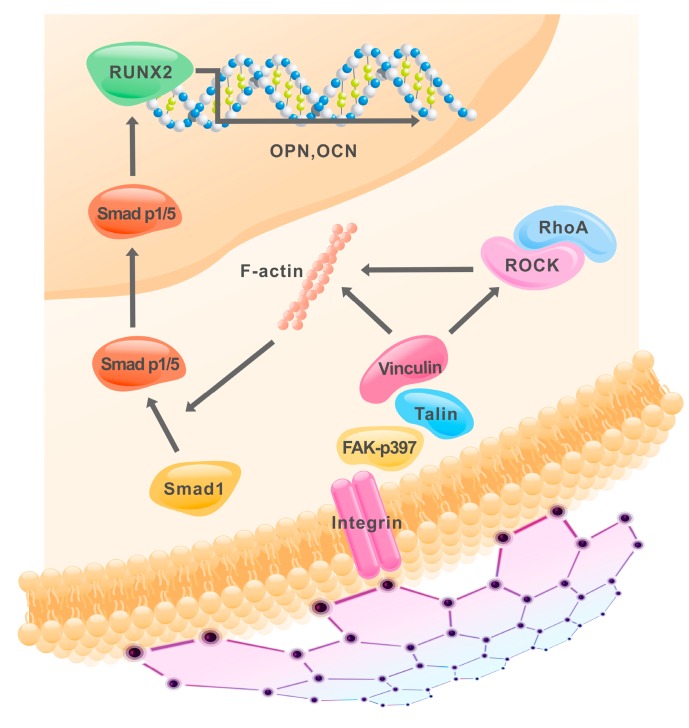
Mono-atomic graphene film promotes osteogenic differentiation of human MSCs via activation the integrin-FAK axis.

Despite our provocative findings showing osteogenic induction by CVD-grown graphene in vivo and elucidating an underlying mechanotransduction-related signaling cascade, this work has limitations. For instance, PDMS with tunable properties has been shown to induce “inside-out” changes in cells to influence migration, stiffness and differentiation via adjusting the physical characteristics of the microenvironment [18,21]. Moreover, our synthetic material and the two-dimensional set-up of the experiments do not mimic the complex organic three-dimensional environments in which cells are imbedded in vivo. In addition, the contribution of the integrin-FAX axis in the graphene-induced osteogenic differentiation was elucidated in vitro, rather than in the animal. In addition, the osteogenic potential was evaluated in an ectopic model. Future studies shall evaluate the potential of graphene scaffold in situ and benchmark its potential to induce osteogenesis to other materials used for bone tissue engineering research. On other hand, these caveats provide us future avenues for investigation. Future studies will investigate the contribution of other integrin-relevant links (e.g., serine/threonine kinase and Rac pathways) and pathways involved in the differentiation of stem cells into osteoblasts (e.g., TGF-β/SMAD, Wnt and BMP-2). Despite of these limitations, our work provides the evidence that graphene induces the osteogenic differentiation by the upregulation of proteins sensitive to physical stimuli involved in the integrin-FAK axis. Thus, the molecular properties of graphene are capable of activating the integrin-FAK transmembrane complex that recruit the activity of both ROCK1 and F-actin that, in turn, stimulate the phosphorylation of Smad p1/5, upstream of RUNX2, OPN and OCN expression to bring osteogenesis closer to fruition (Figure 6).

## 4. Material and Methods

### 4.1. Graphene Films and Scaffold Preparation

Graphene samples were produced by CVD using a custom-built furnace in a Class 1000 clean room facility at the NUS Centre for Advanced 2D Materials and Graphene Research Centre.

For single-layer graphene films, copper foils (Graphene Platform, Tokyo, Japan) were placed in a quartz tube. Thereafter, hydrogen gas (8 sccm) was introduced into the chamber and the temperature was increased to 1010 °C (25 °C/min) and maintained for 35 min. The graphene films were grown by flowing 16 sccm of CH_4_ into the tube for 30 min at 500 mTorr. Finally, the tube was cooled to room temperature with H_2_. The graphene-coated copper foils were spin-coated with polymethyl methacrylate (PMMA, Sigma, Saint Louis, MO, USA) and maintained at 180 °C for 10 min. The uncoated surface of the copper foil was etched with oxygen plasma (3 min, 50 W, 50 sccm, Vita-mini reactive ion etching, FEMTO Science Inc., Gyeonggi-Do, Korea). Following this, the copper was etched with 1.5% ammonium persulphate solution for 8 h and the graphene/PMMA transferred to deionized water for 2 h. Thereafter, the floating membrane was gently scooped onto the target polydimethylsiloxane (PDMS) substrates and the PMMA dissolved in acetone for 12 h. Finally, the graphene-coated samples were washed in isopropyl alcohol for 3 h.

The graphene scaffold was grown using nickel templates (density 320 ± 25 g/m^2^, pore size ~500 μm, Alantum Advanced Technology Materials, Seongnam, Korea) with the same CVD protocol. Thereafter, the nickel was removed with FeCl_3_ solution for 72 h at room temperature and washed with deionized water for 72 h.

**Figure 7:**
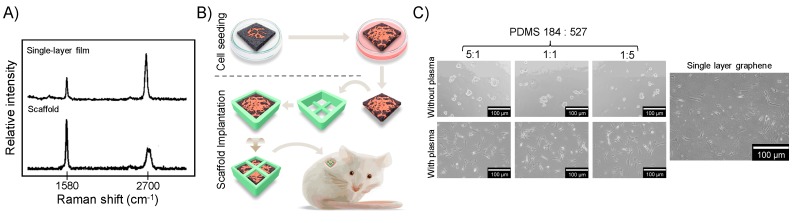
(**A**) Raman characterization. The Raman fingerprints confirmed the successful production of single layer graphene films and multilayer graphene scaffolds. (**B**) Graphene scaffold transplantation in vivo. MSC were seeded in the chemical vapor deposition (CVD)-grown graphene scaffolds (10 × 10 × 1 mm) and maintained in suspension plates at 37 °C for seven days to allow for attachment and proliferation. The scaffolds were placed in three-dimensionally (3D)-printed polylactide protection containers and implanted subcutaneously into immunodeficient mice for four weeks. (**C**) Cell attachment on PDMS with tunable elastic properties and graphene. Cells attached and proliferated on the surface of the oxygen plasma-etched PDMS substrates and single layer CVD-grown graphene.

Raman spectra of graphene films and scaffolds were obtained at room temperature, with an excitation laser source of 532 nm and power of 0.1 mW (Raman Microscope CRM 200, Witec, Ulm, Germany) and confirmed the production of single-layer films and multi-layer graphene scaffolds (Figure 7A).

### 4.2. Graphene Scaffold Transplantation In Vivo

The use of human cells and animals were approved by the NUS Institutional Review Board, Institutional Biosafety Committee and Institutional Animal Care and Use Committee (R17-0956, 12/12/2017).

Human dental pulp stem cells (DP003F, Alcells, Alameda, USA) were cultured in basal growth culture medium ((Dulbecco’s modified Eagle’s medium (DMEM, Invitrogen, Carlsbad, CA, USA), 10% fetal bovine serum (Invitrogen) and 1% penicillin/streptomycin (Invitrogen)) until 70~80% confluence and harvested (TrypLE, Invitrogen). As shown in Figure 7B, MSC (2 × 10^4^, passage 3) were seeded in the graphene scaffold (0.8 × 0.8 × 0.2 cm) and maintained undisturbed for seven days. Following that, the scaffolds (*n* = 6) were placed inside 3D-printed polylactide protection containers (Cube, 3D Systems, Columbia, SC, USA) and transplanted into the subcutaneous space of immunodeficient mice (CB-17 SCID, Invivos, Singapore). After 28 days, specimens were retrieved, fixed with 4% formaldehyde solution in phosphate-buffered saline at 4 °C for 24 h and stained with hematoxylin and eosin (H&E). Immunohistochemical analyses of the tissues formed were performed for OPN and RUNX2 (1:1000, Abcam, Cambridge, UK), and anti-human mitochondria antibodies (1:500, Abcam). Controls were tissue sections stained with an isotype-matched non-specific IgG antibody.

### 4.3. Role of Integrin-FAK Axis in the Graphene-Induced Osteogenic Differentiation

#### 4.3.1. Substrate Preparation

To create the PDMS substrates with tunable modulus of elasticity, we blended Sylgard 184 and 527 (Dow Corning, Midland, TX, USA) in different proportions (5:1 = 1.3 MPa, 1:1 = 830 kPa, and 1:5 = 130 kPa [41]). Glass slides (22 mm × 22 mm) were covered with the freshly mixed PDMS and spin-coated (2000 rpm, 90 s). The PDMS substrates were maintained at 120 °C for 1 h followed by 60 °C overnight. To improve cell attachment, the PDMS substrates were treated with oxygen plasma (3 min, 5 W, 20 sccm, Vita-mini reactive ion etching, Figure 7C). Thereafter, half of the PDMS substrates were coated with a single layer of graphene (Gp) by the scooping method.

#### 4.3.2. Inhibitor Concentration Optimization

Echistatin, Y27632 and DMH1 were used to selectively inhibit the expression of key proteins expressed in the integrin-FAK axis namely integrin, Rho-associated kinase 1 (ROCK1) and Smad.

To optimize the concentration of the inhibitors, MSC were seeded in 96-well flat-bottom plate (800/well) and maintained undisturbed in basal growth culture medium for 24 h. Thereafter, the culture medium was completely removed and 100 μL of DMEM with different concentrations of the inhibitors were added and changed every 48 h. Cell proliferation was evaluated for seven days by an MTS assay (CellTiter 96 AQueous One Solution Assay, Promega, Madison, WI, USA). The inhibitory ratio of cell proliferation was calculated based on the absorbance at 490 nm (Multiskan GO Microplate Spectrophotometer, Thermo Fisher Scientific, Waltham, MA, USA) obtained for the untreated cells (control). Statistical analyses were performed with Mann–Whitney test (α = 5%, SPSS Statistics 22, IBM, Armonk, NY, USA). The concentrations that inhibited cell proliferation by ~30% were selected for the subsequent tests.

#### 4.3.3. Role of Integrin-FAK Axis on Graphene Osteogenic Differentiation

MSC (7 × 10^3^) were seeded on the PDMS substrates alone (5:1, 1:1 and 5:1) or coated with graphene (Gp) and cultured for 10 days. Thereafter, the expression of integrin-FAK axis-related (FAK-p397, F-actin, ROCK, Smad p1/5) and bone-related proteins (RUNX2, OCN and OPN) were assessed by Western blot (all antibodies from Abcam) and gels imaged using ChemiDoc MP Imaging System (Bio-Rad, Hercules, CA, USA). Cells cultured with echistatin (10 nM), Y27632 (50 μM) or DMH1 (50 μM) were the controls. Three independent samples were evaluated per group. The intensity of the bands was quantified with ImageJ (NIH, Bethesfa, MD, USA). For the relative quantification, we firstly confirmed that the housekeeping protein (GAPDH) was constant across the groups. Secondly, we obtained the lane normalization factor (LNF) by dividing the housekeeping bands on the blot by the highest signal for the housekeeping protein identified. The NFs were approximately 1.0 for all groups indicating homogeneous housekeeping protein expression. Thirdly, the protein density (PD) of all the proteins of interest (e.g., FAK) was normalized to their respective housekeeping protein in each lane. Finally, the normalized signal of each PD was divided by the LNF and the relative expression was calculated as [(Gp − PDMS)/PDMS] × 100.

## 5. Conclusions

CVD-grown graphene induces osteogenic differentiation of MSCs due to its molecular and physical properties (e.g., elastic properties, surface features) without the assistance of the conventional cocktail of osteogenic inducers considered necessary in other platforms. Here, we extended these findings by showing that graphene scaffolds also promote the osteogenic differentiation of MSCs in vivo. Osteogenesis was corroborated with the positive expression of RUNX2 and OPN, classical markers for osteogenic differentiation. Complementary in vitro tests demonstrated that the osteogenic differentiation was mediated by the activation of the mechanosensitive integrin-FAK axis. These developmental attributes appear to be inherent to the mono-atomic graphene sheet as changing the elastic modulus of the underlying PDMS had little effect on their ability to induce osteogenesis. Our findings deepen the understanding of the effects of graphene on biological systems and broaden the potential avenues for the use of this single-atom thin material to promote osteogenesis without the delivery limitations of exogenous chemical inducers.
